# Inpatient morbidity and structural care burden in oropharyngeal carcinoma in Germany: a nationwide cross-institutional EHR analysis using an AI-enabled data network

**DOI:** 10.1007/s00405-026-10583-4

**Published:** 2026-09-17

**Authors:** Sabine Eichhorn, Anne Stöckert, Franz Niklas Mitze, Maximilian Bucksch, Eren Erdogan, Inga Marte Charlott Seuthe, Mehdi Dastur, Jonas Jae-Hyun Park

**Affiliations:** 1 Department of Otorhinolaryngology and Head and Neck Surgery, University of Witten/Herdecke, St. Josefs-Hospital, Hagen, Germany; 2 Tiplu GmbH, Hamburg, Berlin Germany

**Keywords:** Oropharyngeal neoplasms, Hospitalization, Microvascular reconstruction, Health services research, Electronic health records, Artificial Intelligence

## Abstract

**Purpose:**

This study aims to characterize hospitalization-level morbidity and care burden associated with oropharyngeal carcinoma (OPC) in Germany and assess the contribution of terminology-based extraction from narrative electronic health record (EHR) documentation beyond structured administrative coding.

**Methods:**

This nationwide retrospective observational study analyzed inpatient data from 96 German tertiary care hospitals (2016–2022). OPC hospitalizations were identified using ICD-10-GM principal diagnosis codes. Treatment patterns, morbidity indicators, and resource utilization measures were derived from OPS procedural codes, structured clinical variables, and terminology-based extraction from predefined narrative EHR sections. Analyses were conducted at the hospitalization level.

**Results:**

Among 13,925 OPC hospitalizations, major pharyngeal resections were documented in 9.0% of cases. The proportion reconstructed with microvascular free flaps more than doubled, while resections without reconstruction declined. Robotic-assisted surgery was documented in 0.1% of OPC hospitalizations. Mechanical ventilation was documented in 3.7% of cases, and 19.7% had documentation indicative of tracheostomy. Median hemoglobin decreased during hospitalization, and documented RBC transfusion use increased descriptively from 5.4% in 2016 to 7.6% in 2022. The in-hospital mortality rate was 2.8%. Terminology-based EHR extraction identified additional documentation of selected comorbidities and morbidity markers beyond structured coding alone.

**Conclusion:**

Hospitalization-level OPC care in German tertiary hospitals involves substantial inpatient care burden, including complex reconstruction, airway-related morbidity, and physiologic morbidity markers. Combining structured administrative data with terminology-based extraction from narrative EHR documentation provided additional routine-data markers beyond coding alone. These findings should be interpreted as complementary hospitalization-level information rather than as validated clinical endpoints or evidence of causal treatment effects.

**Supplementary Information:**

The online version contains supplementary material available at https://doi.org/10.1007/s00405-026-10583-4.

## Introduction

Oropharyngeal carcinoma (OPC) represents a major subgroup of head and neck malignancies and remains a substantial cause of morbidity and mortality in otorhinolaryngological practice. Over recent decades, OPC incidence has increased in many European countries, largely attributed to cohort effects and the rising prevalence of human papillomavirus (HPV)–associated tumors [1, 2]. In Germany, population-based cancer registry data demonstrated pronounced regional variation and an overall upward trend in OPC incidence [3].

Management of OPC typically requires multimodal treatment strategies, including surgery, radiotherapy, and systemic therapy, either alone or in combination. While these strategies have improved oncologic outcomes, they are frequently associated with considerable inpatient morbidity, particularly in patients undergoing extensive resections or requiring intensive peritherapeutic management [4].

In Germany, epidemiological and health services research on OPC has predominantly relied on administrative data sources, such as nationwide hospital statistics from the Federal Statistical Office (DESTATIS), cancer registry data consolidated by the German Centre for Cancer Registry Data, and routine datasets from statutory health insurance providers [4–6]. Although these sources enable large-scale analyses of incidence and treatment patterns, they are largely based on the German Modification of the International Classification of Diseases, 10th Revision (ICD-10-GM) and the German Operation and Procedure Classification System (OPS) coding systems linked to the German Diagnosis-Related Groups (DRG) reimbursement framework. Consequently, they capture diagnoses and procedures relevant for billing but only incompletely reflect clinically nuanced indicators of inpatient morbidity.

Clinically relevant morbidity markers, such as perioperative hemoglobin decline, transfusion requirements, airway management, duration of mechanical ventilation, and postoperative complications, are often underrepresented in administrative coding or recorded in unspecific categories. Furthermore, coding practices may be influenced by reimbursement structures, potentially introducing systematic distortions when administrative data are used to estimate clinical burden [7].

Electronic health records (EHRs) contain extensive narrative clinical documentation generated during routine care and their use for epidemiological research has long been discussed, both with considerable optimism regarding their potential and with justified caution concerning data quality, heterogeneity, and methodological challenges [8, 9]. Advances in terminology-based, semantic analysis allow structured extraction of clinically meaningful variables from such unstructured text. In head and neck oncology, this approach offers the opportunity to complement administrative data with semantically enriched clinical information and thereby improve characterization of inpatient morbidity.

The present study analyzes a large nationwide inpatient cohort from Germany integrating administrative coding with variables derived from narrative EHR documentation through terminology-based extraction. The aim was to characterize the inpatient morbidity burden associated with OPC hospitalizations and to assess whether terminology-based extraction provides additional clinically relevant information beyond ICD- and OPS-based data.

## Materials and methods

### Study design and data infrastructure

This non-interventional, retrospective observational study was conducted as a secondary analysis of routinely collected inpatient data from 96 German tertiary care hospitals, constituting a cross-institutional cohort within a nationwide AI-enabled data network. The network has previously been evaluated and shown to broadly reflect the German inpatient population [10].

The dataset, provided by Tiplu GmbH (Hamburg, Germany), included anonymized administrative and clinical documentation collected between January 2016 and December 2022. All data were anonymized prior to analysis. The study was conducted in accordance with the Declaration of Helsinki and was approved by the Ethics Committee of Witten/Herdecke University (S-140/2022).

### Data harmonization

Data harmonization used a pre-existing operational infrastructure implemented independently of the present study. Local data structures, source fields, document types, and storage locations were mapped to a common standardized patient record model according to centrally defined specifications. Thus, although the underlying hospital information systems differed between institutions, the same target model, variable definitions, and mapping principles were applied across all sites.

Diagnoses and procedures were represented using ICD-10-GM and OPS, laboratory measurements were standardized using Logical Observation Identifiers Names and Codes (LOINC), medication information using the Anatomical Therapeutic Chemical classification system (ATC), and clinical document types using the German Clinical Document Class List (KDL). This enabled identical cohort definitions and analytical rules across hospitals and supported rule-based terminology searches within predefined document categories. Data processing was distributed; patient-level raw data remained within each hospital, and only aggregated anonymized data were analyzed centrally. The mappings were not developed or formally validated specifically for this study, and residual differences in documentation completeness and local implementation cannot be excluded.

### Cohort definition

OPC hospitalizations were identified using the ICD-10-GM codes for malignant neoplasms of the oropharynx and adjacent subsites. For descriptive presentation, the principal-diagnosis codes were grouped into six anatomical subsite categories: base of tongue and vallecula (C01, C02.4, and C10.0); palate (C05.1, C05.2, C05.8, C05.9, and C09.1); tonsil and lateral wall of the oropharynx (C09.0, C09.8, C09.9, and C10.2); posterior wall of the oropharynx (C10.3); overlapping sites of the oropharynx (C10.8); and other or unspecified oropharyngeal and adjacent pharyngeal sites (C10.9, C14.0, C14.2, and C14.8). The C14 codes were retained to account for routine coding of tumors assigned to nonspecific, overlapping, or adjacent pharyngeal locations. The grouped subsite distribution was assessed descriptively; treatment patterns and morbidity indicators were not stratified by individual diagnosis code or subsite group. Only hospitalizations with these codes recorded as the principal diagnosis were included.

All analyses were conducted at the level of individual hospitalizations. Throughout the manuscript, the terms “case” and “hospitalization” are used synonymously and refer to a single inpatient hospital episode. Longitudinal linkage of repeated admissions belonging to the same individual was not possible; therefore, results represent hospitalization-based estimates rather than patient-level incidence or treatment trajectories.

### Outcomes

The primary objective of this study was to characterize the epidemiological profile and inpatient care burden associated with hospitalizations for OPC in German hospitals. Accordingly, inpatient morbidity indicators, resource utilization measures, and in-hospital mortality were defined as primary outcome measures.

The secondary objective was to assess the incremental contribution of terminology-based EHR data extraction compared with structured administrative coding in identifying clinically relevant variables within routine inpatient data.

All outcome measures were analyzed at the hospitalization level.

### Variables

#### Treatment-related variables

Treatment-related variables included major surgical tumor resection, neck dissection, radiotherapy, and systemic antineoplastic therapy. Major surgical resections were identified using OPS codes 5-295 and 5-296, and neck dissections using codes 5-403. Radiotherapy and systemic antineoplastic therapy were identified using codes 8–52 and 8–54, respectively.

Peritherapeutic events comprised complex intensive care treatment (OPS 8-980), transfusion of cellular blood components (OPS 8-800), and surgical closure of a pharyngocutaneous fistula (OPS 5-294.1). Newly performed tracheostomies during the index hospitalization were identified using OPS codes 5-311 and 5-312. The presence of a tracheostoma, irrespective of whether it was pre-existing or newly created, was identified using ICD-10-GM codes Z93.0, Z43.0, J95.0, and R13.1. R13.1 was included because its definition specifically covers dysphagia associated with a suction-dependent tracheostoma and a tracheal cannula with a partially or fully inflated cuff. Each hospitalization was counted only once, irrespective of the number or combination of qualifying ICD-10-GM codes documented.

#### Comorbidities and substance-related risk factors

Arterial hypertension, diabetes mellitus, overweight- or obesity-related documentation, and nicotine- or tobacco-use-related documentation were identified by combining structured ICD-10-GM diagnoses with terminology-based extraction from narrative EHR documentation. The proportions identified exclusively through terminology-based extraction were additionally assessed.

An exploratory terminology-based search for alcohol-related documentation was additionally performed using predefined alcohol-related search concepts. Alcohol withdrawal syndrome with delirium was analyzed separately and identified exclusively using ICD-10-GM code F10.4.

#### Morbidity indicators

Indicators of inpatient morbidity included hemoglobin levels at admission and nadir during hospitalization, transfusion of red blood cell (RBC) concentrates, duration of mechanical ventilation, length of hospital stay, and documented complications.

Hemoglobin values, duration of mechanical ventilation, and length of hospital stay were extracted as structured variables from hospital information systems. Transfusion of red blood cell (RBC) concentrates was identified using OPS code 8-800.c. Documented complications were identified using relevant OPS codes and information extracted from narrative EHR documentation using terminology-based methods.

### Data sources and measurement

Diagnoses were identified using ICD-10-GM coding. Structured clinical variables were extracted directly from hospital information systems. Procedure-related variables were derived from OPS codes in accordance with the German coding rules applicable during the study period. For the selected procedures, codes were generally recorded and counted once per hospitalization. Radiotherapy was an exception, as the corresponding OPS codes represent individual treatment sessions and may therefore be recorded multiple times during a single inpatient stay. Accordingly, non-radiotherapy procedures were analyzed at the hospitalization level, whereas radiotherapy was additionally reported as the number of coded treatment events. Neither measure represents the number of unique patients, as repeated hospitalizations could not be linked longitudinally.

Variables not reliably captured through structured data were additionally assessed using terminology-based semantic search applied to predefined narrative EHR sections, including discharge summaries, operative reports, radiology reports, and pathology reports. For each variable, predefined search concepts and synonyms where applicable were used. The routine extraction engine additionally applied approximate string matching based on Levenshtein distance to capture minor spelling and word-form variants. The terminology-based search did not include automated negation detection. To minimize false-positive retrievals, search queries were restricted to document sections where affirmative use of the respective terms was most probable.

During development and routine application of the extraction approach, selected outputs were manually reviewed for clinical plausibility as a quality-control measure. These reviews did not constitute a formal validation. The terminology-based extraction was not validated against an independently annotated reference standard, either for the general extraction approach or specifically for the present study. Accordingly, terminology-based variables were considered unvalidated documentation-based markers and not validated clinical endpoints.

The exact ICD-10-GM and OPS codes, terminology search concepts, and EHR document sections used for the key variables are provided in Supplementary Table S1.

### Bias and limitations

Potential bias arose from ICD-10-GM–based case identification, inter-hospital variation in coding and documentation, temporal changes in clinical practice, and incomplete or misclassified terminology-based extraction, which lacked automated negation and contextual assertion detection. Repeated admissions could not be linked, and tumor stage, HPV status, and treatment intent were unavailable. Analyses were therefore restricted to descriptive hospitalization-level outcomes and were not designed to assess treatment effects. Standardized coding frameworks, harmonized data structures, predefined document sections, and cautious interpretation were used to reduce potential bias.

### Study size and statistical analysis

The study size was determined by the availability of routine inpatient data from participating hospitals during the observation period. All hospitalizations meeting the predefined inclusion criteria were included; no a priori sample size calculation was performed.

Descriptive statistics were used to summarize categorical variables as absolute and relative frequencies and continuous variables using means, medians, quartiles, minimum and maximum values, and standard deviations. Top- and bottom-coded values for age, length of stay, duration of mechanical ventilation, and hemoglobin levels were recoded using predefined numeric approximations for descriptive analyses and were not treated as missing. Missing values were reported descriptively without imputation.

Given the descriptive aim of the study, no inferential statistical testing was performed. The analysis was not designed to test predefined hypotheses, compare treatment groups, or estimate causal associations. Meaningful risk-adjusted comparisons were further limited by the absence of patient-level linkage and key clinical covariates. Moreover, given the large number of hospitalizations, conventional significance testing could identify small absolute differences as statistically significant without necessarily indicating clinical relevance. Absolute frequencies, proportions, and distributional measures were therefore considered most appropriate for the intended descriptive characterization. Temporal changes are presented descriptively and should not be interpreted as statistically confirmed trends.

All analyses were conducted using R statistical software, version 4.5.2.

## Results

### Cohort characteristics

Among 8,277,398 inpatient cases from 96 hospitals during 2016–2022, a total of 46,364 were identified with a malignant head and neck tumor, of which 13,925 (30.0%) met the predefined criteria for oropharyngeal carcinoma.

The most frequent grouped principal-diagnosis categories were overlapping sites of the oropharynx (*n* = 4,164; 29.9%), the tonsil and lateral wall of the oropharynx (*n* = 4,007; 28.8%), and the base of tongue and vallecula (*n* = 3,398; 24.4%). The remaining hospitalizations involved the palate (*n* = 1,222; 8.8%), other or unspecified oropharyngeal and adjacent pharyngeal sites (*n* = 844; 6.1%), or the posterior wall of the oropharynx (*n* = 290; 2.1%) (Supplementary Table S2).

The median age of the OPC cohort was 63.0 years (IQR 57.0-69.9), and male sex was documented in 73.2% of cases.

### Treatment patterns

#### Surgical management

A partial or radical pharyngeal resection was performed in 1,252 cases (9.0%) of all hospital cases with oropharyngeal carcinoma. The annual number of pharyngeal resections remained largely stable over the observation period. The transoral approach was used most frequently, accounting for 64.4%, followed by pharyngotomy in 23.6% of cases.

**Fig. 1:**
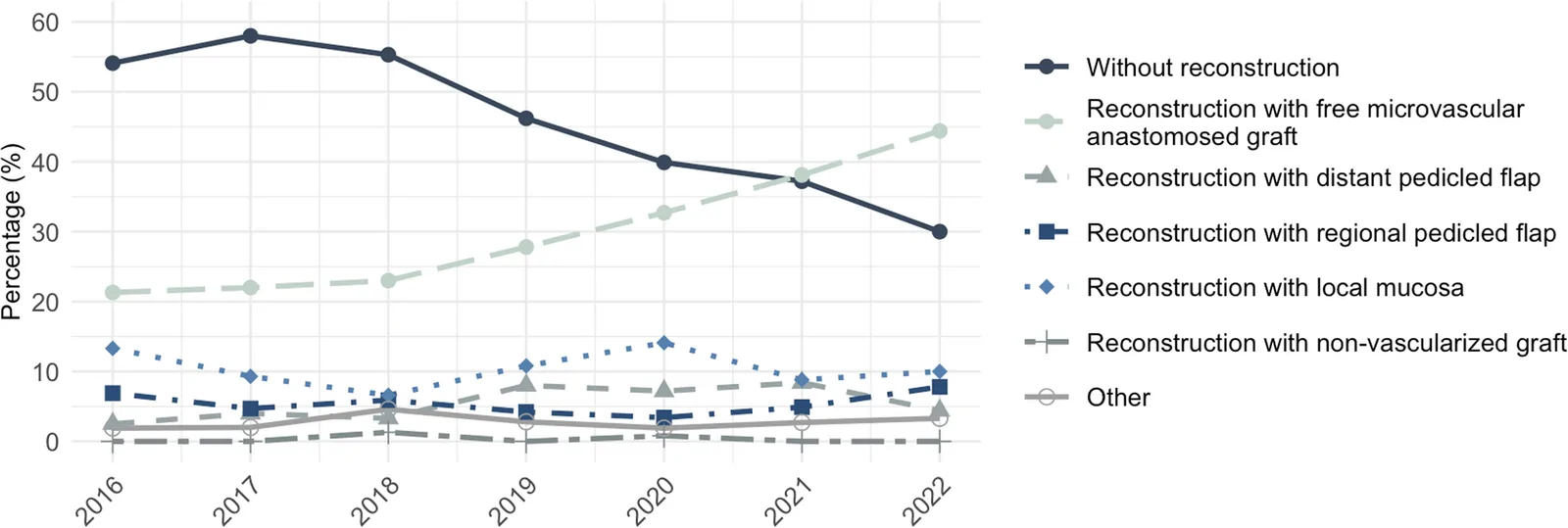
Temporal trends in reconstruction techniques after partial or total Pharyngectomy for Oropharyngeal cancer

In 46.6% of these procedures, no reconstruction was performed; however, this proportion declined substantially over time, decreasing from 54.1% in 2016 to 30.0% in 2022. In contrast, the use of microvascular free flaps for reconstruction increased steadily throughout the study period. Overall, microvascular free flaps were utilized in 29.8% of resections, with their proportion rising from 21.3% in 2016 to 44.4% in 2022. Meanwhile, the share of reconstructions performed using local or regional flaps remained stable over the years (Fig. 1).

Pharyngoplasty without preceding partial or radical pharyngeal resection was documented in 74 cases (0.5%) of the OPC cohort. Among these interventions, reconstruction was performed using microvascular free flaps in 48.7% of cases, pedicled myocutaneous flaps in 18.9%, and local mucosal reconstruction in 25.7%.

CO₂ laser use was documented in 6.8% of OPC cases, occurring most frequently in tonsillar tumors (32.7%) and tumors of the base of tongue (27.8%).

Robotic assistance was documented in 0.1% of OPC cases, most commonly for tumors of the base of tongue (42.1%) and the tonsillar region (15.8%).

Neck dissection–related procedures were documented in 2,128 OPC cases (15.3%), comprising selective neck dissections (80.9%) and radical or modified radical neck dissections (4.6%), and the distribution remained stable over the observation period.

#### Non-surgical treatments

Radiotherapy-related treatment events totaled 5,486. The vast majority (97.8%) consisted of high-voltage external beam radiotherapy, whereas 2.0% represented brachytherapy and 0.1% proton therapy. As radiotherapy OPS codes represent individual treatment sessions, multiple events could be documented within a single hospitalization. These counts therefore reflect inpatient radiotherapy activity and not the number of treated patients or complete treatment courses.

**Fig. 2:**
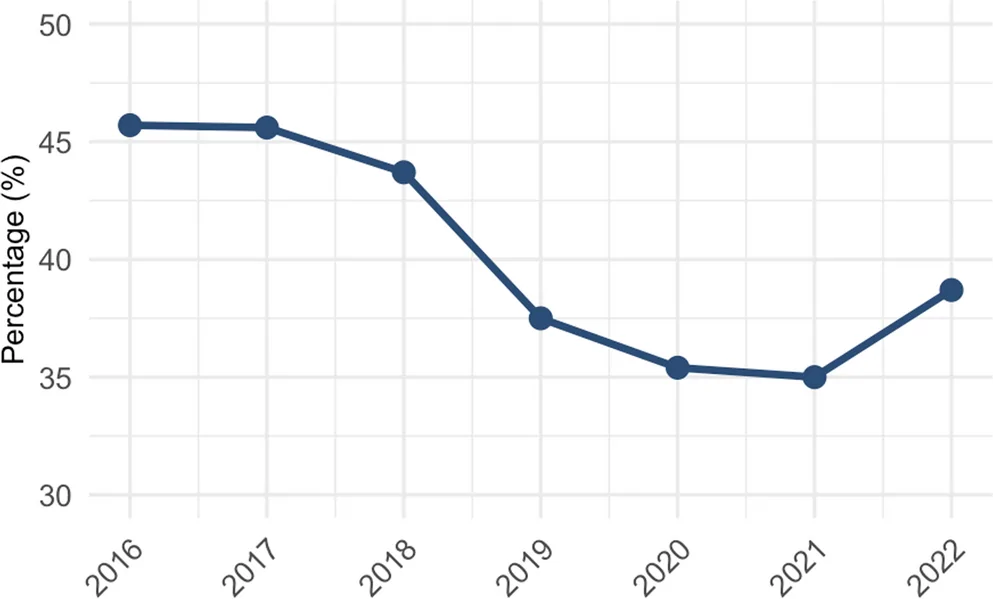
Temporal trends in inpatient radiotherapy for Oropharyngeal carcinoma

The distribution of radiotherapy modalities remained largely stable over time, while the proportion of hospitalizations with documented radiotherapy declined from 2016 onward, reaching its lowest level in 2021 before partially recovering in the final study year (Fig. 2).

**Fig. 3:**
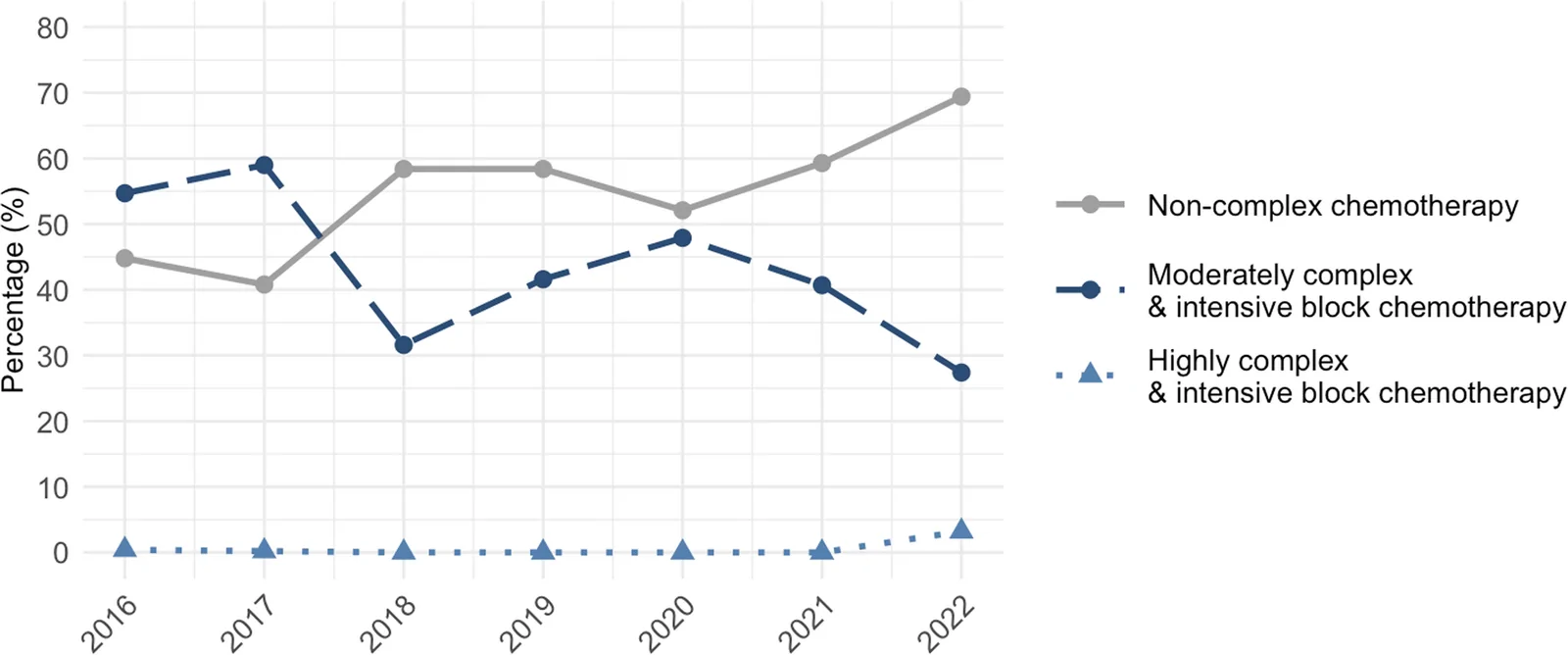
Temporal trends in chemotherapy regimen complexity among hospitalized cases with Oropharyngeal carcinoma

Chemotherapy-related procedures were documented in 3,553 cases (25.5%). Non-complex regimens accounted for 55.8% of these cases, moderately complex regimens for 43.9%, and highly complex regimens for 0.4%. Over the observation period, the proportion of moderately complex regimens declined markedly, decreasing from 54.7% in 2016 to 27.4% in 2022. In contrast, the share of non-complex regimens increased steadily, rising from 44.8% in 2016 to 69.4% in 2022 (Fig. 3). No electrochemotherapy procedures were documented during the study period.

**Fig. 4:**
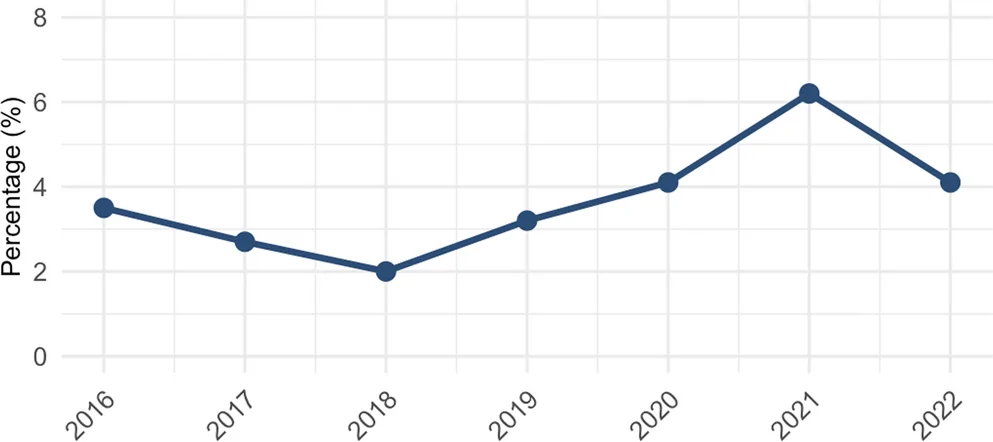
Temporal trends in inpatient immunomodulatory or antibody-based therapies for Oropharyngeal carcinoma

OPS-coded immunomodulatory or antibody-based therapies were documented in 544 OPC hospitalizations (3.9%). Of these, 75.6% involved non-modified monoclonal antibodies and 23.5% modified antibody-based therapies. Annual proportions declined until 2018, increased to a peak of 6.2% in 2021, and decreased to 4.1% in 2022 (Fig. 4).

### Comorbidities and substance-related risk factors

The median number of secondary diagnoses per OPC hospitalization was 6 (IQR 3–11; range 1–20), with a mean of 7.5.

The combined use of ICD-10-GM coding and terminology-based EHR analysis identified arterial hypertension in 44.5% of cases, diabetes mellitus in 12.7%, and overweight- or obesity-related documentation in 4.5%.

Among substance-related factors, nicotine- or tobacco-use-related documentation was identified in 14.6% of hospitalizations using the combined approach. Alcohol-related documentation identified through exploratory terminology-based extraction was present in 0.1% of hospitalizations. Alcohol withdrawal syndrome with delirium, identified exclusively using ICD-10-GM code F10.4, was documented as a secondary diagnosis in 0.2% of hospitalizations.

### Treatment-associated inpatient morbidity indicators

#### Duration of mechanical ventilation

Mechanical ventilation was documented in 522 cases (3.7%). Among these, ventilation lasted up to 24 h in 26.2%, 24–72 h in 47.3%, and more than 72 h in 26.4%. The median duration of mechanical ventilation was 48 h (IQR 22–77).

#### Complex intensive care treatment

Complex intensive care treatment was identified in 145 cases (1.0%). Among these, 68.3% were assigned to OPS-defined point ranges of 1–184 points, 17.9% to 185–552 points, and 7.6% to 553–1,104 points. The remaining 6.2% of cases were associated with OPS-defined point ranges exceeding 1,104 points.

#### Tracheostomy

Tracheostomy-related documentation was present in 2,743 OPC hospitalizations (19.7%), irrespective of whether it was pre-existing or newly created. An OPS-coded tracheostomy newly performed during the index hospitalization was identified in 1,253 cases, corresponding to 9.0% of all OPC hospitalizations.

### Flap necrosis, postinterventional bleeding, and surgical closure of a pharyngocutaneous fistula

Flap necrosis was identified in 84 cases (0.6%) of the OPC cohort using a combined approach of ICD-10-GM coding (T86.51) and terminology-based EHR analysis.

Postinterventional bleeding was documented in 1,713 cases (12.3%) based on ICD-10-GM code T81.0 and terminology-based extraction from narrative documentation. As this composite marker was not limited to major tumor resections, it should not be interpreted as a procedure-specific postoperative hemorrhage rate.

In contrast, pharyngeal fistula closure procedures were identified exclusively via procedural coding. Across the observation period, OPS code 5-294.1 was documented in fewer than 0.1% of OPC hospitalizations.

#### Hemoglobin levels during hospitalization and RBC transfusions

Hemoglobin trajectories were assessed within individual hospitalizations, whereas annual proportions of hospitalizations with documented RBC transfusion were compared descriptively across the study period.

The median baseline hemoglobin level of all OPC cases was 12.5 g/dL (IQR 11.5–14.5), with a mean of 12.7 g/dL. During hospitalization, the median hemoglobin nadir was 11.5 g/dL (IQR 9.5–13.5; mean 11.7 g/dL), corresponding to decreases of 1.0 g/dL for the median and 0.94 g/dL for the mean relative to baseline values. Differences at the 25th and 75th percentiles were 2.0 g/dL and 1.0 g/dL, respectively.

**Fig. 5:**
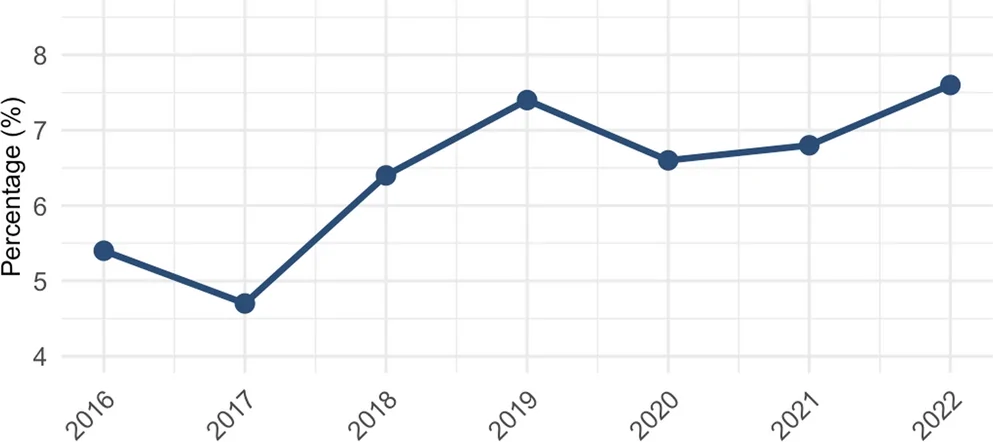
Temporal trends in inpatient RBC transfusions for Oropharyngeal carcinoma

Overall, RBC transfusion was documented in 6.5% of OPC hospitalizations, increasing descriptively from 5.4% in 2016 to 7.6% in 2022 (Fig. 5).

#### Length of stay and in-hospital mortality

The median total length of stay was 4 days (IQR, 1–9; mean 7.9 days). Overall, 31.1% of hospitalizations exceeded the DRG-defined mean length of stay, and 9.0% exceeded the upper DRG threshold, with proportions remaining stable over time.

The in-hospital mortality rate was 2.8%.

### Incremental contribution of terminology-based EHR analysis

Terminology-based extraction identified additional documentation beyond structured administrative coding. Among hospitalizations with documented hypertension, 9% were identified exclusively through terminology-based extraction; the corresponding proportions were 11% for diabetes mellitus, 32% for overweight- or obesity-related documentation, 20% for nicotine- or tobacco-use-related documentation.

Among hospitalizations with documented flap necrosis, 44% were identified exclusively through terminology-based extraction. Similarly, 51% of documented postinterventional bleeding events were identified exclusively through terminology-based extraction.

## Discussion

This nationwide cross-institutional analysis characterizes hospitalization-level care and morbidity for OPC in Germany from 2016 to 2022.

### Relation to previous data sources

Previous nationwide analyses of head and neck cancer in Germany have predominantly relied on cancer registries, hospital discharge statistics, or claims data. These sources provide robust information on incidence and treatment patterns but capture clinically nuanced morbidity indicators only incompletely.

By integrating structured administrative data with selected variables derived from narrative EHR documentation, the present study provides a more detailed hospitalization-level characterization of inpatient care. The variables derived through terminology-based extraction should, however, be interpreted as complementary routine-data markers rather than fully validated clinical endpoints.

## Epidemiological relevance and resource utilization

OPC accounted for nearly one third of malignant head and neck tumor hospitalizations. Median length of stay was 4 days; 31.1% of hospitalizations exceeded the DRG-defined mean length of stay, and 9.0% the upper DRG threshold, indicating the relevant inpatient care and resource utilization. The observed in-hospital mortality rate of 2.8% is consistent with previous hospitalization-based studies of head and neck cancer. A German nationwide DRG-based analysis reported an average in-hospital mortality rate of approximately 4% [11], while U.S. studies reported rates of 4.2% for hospitalized head and neck cancer patients [12] and 2.9% for oral and oropharyngeal cancer hospitalizations [13]. Although direct comparisons are limited by differences in patient populations, treatment status, and healthcare systems, our estimate appears plausible within the range of published data.

### Surgical workload and reconstructive practice

Major pharyngeal resections accounted for 9% of all OPC hospitalizations. While the annual number of these resections remained largely stable, the proportion reconstructed with microvascular free flaps more than doubled, and the proportion without reconstruction declined.

Microvascular free flap reconstruction represents a complex and resource-intensive procedure requiring specialized surgical expertise and perioperative care. The observed increase therefore indicates a growing volume of complex reconstructive care within the participating tertiary hospitals. Centralization, changes in referral patterns, case selection, reconstructive preferences, or broader availability of microvascular expertise may have contributed to this development. These factors may explain the observed increase but do not negate the resulting growth in complex reconstructive activity at the tertiary-care level. However, the present data do not demonstrate that the underlying tumors, defects, or individual cases became more complex over time, nor do they permit quantification of workload per center, surgical team, or individual surgeon.

Robotic assistance was documented in only 0.1% of OPC hospitalizations. While this proportion is not directly comparable to patient-level TORS utilization rates because the denominator included all OPC hospitalizations, it is consistent with the limited adoption of TORS in Germany, where high costs and reimbursement barriers have been reported as major obstacles [14]. TORS is an established minimally invasive option for selected patients with OPC, particularly those with early-stage tumors, and has been associated with favorable functional outcomes [15–18].

However, the observed rate may underestimate actual use, as robotic assistance was identified through the supplementary OPS code 5-987, which may not be coded consistently and is not specific to TORS.

### Systemic therapy patterns

While the overall number of chemotherapy-related procedures remained relatively stable over time, the distribution of OPS-defined chemotherapy complexity categories changed during the observation period, with an increasing proportion of non-complex and a decreasing proportion of moderately complex regimens. These categories reflect the administrative OPS classification and do not provide information on individual agents, treatment intent, line of therapy, or clinical indication. The underlying reasons for this change cannot be determined from the present dataset and may include changes in treatment protocols, coding practices, care setting, or patient case mix. Electrochemotherapy has been investigated as a locoregional treatment option for selected patients with oral cavity and oropharyngeal squamous cell carcinoma [19]. However, no electrochemotherapy procedures were documented in the present cohort, precluding conclusions regarding its utilization or clinical role within German inpatient OPC care.

Documentation of OPS-coded immunomodulatory or antibody-based therapies was more frequent from 2019 onward but varied across individual years. Because the OPS categories do not provide drug-level information, the observed pattern cannot be attributed to specific agents, including immune checkpoint inhibitors, or to particular regulatory approvals, indications, or treatment lines.

#### Treatment-associated inpatient morbidity

Markers of inpatient morbidity indicate substantial clinical burden during hospitalization. Mechanical ventilation, OPS-defined complex intensive care treatment, and tracheostomy documentation collectively reflect airway-related and peri-treatment complexity in this population.

Hemoglobin decline and RBC transfusion use provide complementary descriptive markers of inpatient morbidity. Hemoglobin levels decreased between admission and nadir within individual hospitalizations, while the proportion of hospitalizations with documented RBC transfusion increased over the observation period. The available data do not allow determination of whether this temporal change was related to treatment modality, procedural extent, transfusion thresholds, patient case mix, or documentation practices. These findings should therefore be interpreted descriptively and not as evidence of increasing treatment complexity.

The relatively high rate of documented postinterventional bleeding should also be interpreted cautiously. As this composite marker was not restricted to major tumor resections, it does not represent a procedure-specific postoperative hemorrhage rate. Rather, it reflects documented bleeding events across OPC hospitalizations and should be understood as a broad routine-data morbidity marker.

For complications such as postinterventional bleeding and flap necrosis, which may be incompletely captured by structured coding, terminology-based extraction identified additional documentation beyond ICD-10-GM coding alone. While the principal contribution of this study lies in its epidemiological characterization, these findings indicate that administrative data may underestimate clinically nuanced morbidity.

#### Comorbidities and substance-related risk factors

The frequencies of nicotine- or tobacco-use-related documentation and overweight- or obesity-related documentation were lower than population-based estimates [20, 21] and should not be interpreted as prevalence estimates. These variables were derived from routine diagnostic coding and narrative clinical documentation rather than from systematic assessment of smoking status, cumulative tobacco exposure, previous smoking, or body mass index.

Alcohol-related documentation identified through exploratory terminology-based extraction was present in only 0.1% of hospitalizations. The underlying search concepts covered heterogeneous alcohol-related contexts and did not constitute a validated or comprehensive definition of alcohol exposure or alcohol use disorder. The finding should therefore be interpreted as the retrieval yield of the predefined terminology search rather than as a prevalence estimate. Underascertainment due to incomplete documentation or alcohol-related terminology not covered by the search concepts cannot be excluded.

Alcohol withdrawal syndrome with delirium was documented as a secondary diagnosis in 0.2% of OPC hospitalizations. In a recent nationwide US analysis of more than three million patients undergoing major surgery, delirium tremens was documented in 0.2% of cases, while alcohol withdrawal syndrome was associated with increased postoperative complications, prolonged hospitalization, and higher hospital costs; delirium tremens was additionally associated with increased mortality and length of stay [22]. Although differences in healthcare setting, study population, and coding preclude a direct comparison of frequencies, these findings support the potential contribution of alcohol withdrawal delirium to inpatient care burden. Its specific impact on resource utilization could not be assessed in the present study. Importantly, the documented frequency should not be interpreted as a measure of alcohol consumption, harmful alcohol use, or the prevalence of alcohol use.

## Contribution of terminology-based extraction

Terminology-based extraction identified additional documentation of selected comorbidities and complications beyond structured administrative coding alone. This was particularly relevant for variables that may be incompletely captured by ICD-10-GM or OPS coding, such as overweight- or obesity-related documentation, nicotine- or tobacco-use-related documentation, flap necrosis, and postinterventional bleeding. These findings suggest that coding-based analyses alone may underestimate selected clinically relevant morbidity markers.

However, terminology-based extraction cannot recover conditions or exposures that were not documented in the EHR and cannot reliably distinguish true absence from missing or incomplete documentation. As the extraction approach was not formally validated against a study-specific reference standard, variables identified through terminology-based extraction should be interpreted as complementary routine-data markers rather than as validated clinical endpoints.

## Strengths and limitations

Strengths of this study include its large cross-institutional design and the use of a harmonized EHR-based real-world data infrastructure integrating administrative coding with structured and narrative clinical documentation. This approach enables access to physiologic and procedural markers beyond reimbursement-oriented datasets and provides a granular characterization of inpatient morbidity and care intensity.

Several limitations merit consideration. Analyses were conducted at the hospitalization level without longitudinal patient linkage, precluding patient-level inference and reconstruction of complete treatment trajectories. Repeated admissions may therefore reflect either distinct individuals or recurrent care episodes in the same patient.

The underlying data network has previously shown broad concordance with comparable German inpatient hospital statistics, including structurally similar hospital settings. However, because the present analysis was restricted to participating tertiary care hospitals, the findings should primarily be interpreted as reflecting specialized inpatient OPC care. This focus is clinically relevant, as complex head and neck oncologic surgery, microvascular reconstruction, airway management, intensive care, and multimodal treatment are frequently concentrated in tertiary centers. Generalizability to smaller hospitals, less complex inpatient episodes, and outpatient treatment settings remains limited.

Key oncological variables, including tumor stage, HPV/p16 status, treatment intent, recurrence status, and previous therapy, were not available. This is particularly relevant in OPC, as HPV-associated and HPV-negative tumors differ substantially in prognosis, patient characteristics, treatment selection, and treatment response. Consequently, the observed treatment patterns and morbidity indicators could not be stratified by disease biology, stage, or curative versus palliative intent, and temporal changes may partly reflect unmeasured shifts in case mix rather than changes in clinical practice alone.

Case identification and ascertainment of selected outcomes relied on administrative coding and routine clinical documentation, which may be subject to inter-institutional variation, misclassification, and incomplete reporting. Although terminology-based variables were derived from predefined clinically relevant text sections, extraction errors or underdocumentation cannot be excluded. Furthermore, DRG-based length-of-stay benchmarks reflect reimbursement frameworks rather than validated clinical severity scales and should be interpreted accordingly.

Finally, the descriptive design precludes causal inference. The findings should therefore be understood as a characterization of inpatient morbidity burden and structural care complexity rather than as evidence of treatment effects or comparative effectiveness.

### Implications and future directions

The findings provide hospitalization-level information relevant to inpatient resource planning and perioperative care in specialized OPC management, particularly regarding complex reconstructive care, airway-related morbidity, and resource utilization.

Future research integrating longitudinal patient-level linkage, outpatient data, tumor stage, HPV/p16 status, and treatment intent is required to evaluate treatment trajectories and adjusted outcome associations.

## Conclusion

This nationwide hospitalization-level analysis demonstrates substantial inpatient care burden associated with OPC in Germany. Temporal changes in reconstructive practice, particularly the increasing proportion of microvascular free flap reconstruction, indicate a growing volume of complex reconstructive care within participating tertiary hospitals. Hemoglobin decline, documented RBC transfusion use, airway management, and intensive care measures provide additional descriptive markers of inpatient morbidity.

Combining structured administrative data with terminology-based extraction from narrative EHR documentation identified additional routine-data markers beyond coding alone. These findings should be interpreted as complementary hospitalization-level information rather than as validated clinical endpoints or evidence of causal treatment effects.

## Supplementary Information

Below is the link to the electronic supplementary material.

Supplementary Material 1

Supplementary Material 2

## Data Availability

The data underlying this study are not publicly available due to the presence of potentially identifying patient and hospital information and in accordance with ethical and data protection regulations. De-identified aggregated data may be made available upon reasonable request and subject to approval by the responsible data protection authorities. Data access is administered by Tiplu GmbH within the geNuGes project framework.
