# Supplementary material for: Inpatient morbidity and structural care burden in oropharyngeal carcinoma in Germany: a nationwide cross-institutional EHR analysis using an AI-enabled data network
Source: Eur Arch Otorhinolaryngol. 2026 Sep 17;283(12):8121–31. doi: 10.1007/s00405-026-10583-4 (PMC13768223; doi:10.1007/s00405-026-10583-4)
Supplement: Supplementary file 1 [file 405_2026_10583_MOESM1_ESM.docx]

**Supplementary Table S1.** Operational definitions, data sources, and terminology search concepts for key study variables

| **Variable** | **Data source / operational definition** | | | |
| --- | --- | --- | --- | --- |
|  | **ICD-10-GM** | **OPS** | **Hospital information system** | **Terminology-based extraction:** **exact search concepts (German)** |
| **Cohort** | | | | |
| Hospitalizations with malignant neoplasms of the oropharynx and adjacent subsites, recorded as the principal diagnosis | C01, C02.4, C05.1, C05.2, C05.8, C05.9, C09.0, C09.1, C09.8, C09.9, C10.0, C10.2, C10.3, C10.8, C10.9, C14.0, C14.2, C14.8 |  |  |  |
| Age |  |  | Direct extraction |  |
| Sex |  |  | Direct extraction |  |
| **Treatment-related variables** | | | | |
| Major surgical resections |  | 5-295, 5-296 |  |  |
| Plastic-surgical reconstructions |  | Local flaps and free skin grafts: 5-293.0, 5-293.3, 5-295.01, 5-295.11, 5-295.21, 5-295.31, 5-295.x1, 5-296.01, 5-296.11, 5-296.21, 5-296.31, 5-296.x1  Regional pedicled flaps: 5-293.1, 5-295.02, 5-295.05, 5-295.12, 5-295.15, 5-295.22, 5-295.25, 5-295.32, 5-295.35, 5-295.x2, 5-295.x5, 5-296.02, 5-296.05, 5-296.15, 5-296.22, 5-296.25, 5-296.32, 5-296.35, 5-296.x2, 5-296.x5  Free microvascular flaps: 5-293.2, 5-293.4, 5-293.5, 5-295.04, 5-295.14, 5-295.24, 5-295.34, 5-295.x4, 5-296.04, 5-296.06, 5-296.07, 5-296.14, 5-296.16, 5-296.17, 5-296.24, 5-296.26, 5-296.27, 5-296.34, 5-296.36, 5-296.37, 5-296.x4, 5-296.x6, 5-296.x7 |  |  |
| Pharyngoplasty |  | 5-293 |  |  |
| Neck dissection |  | 5-403 |  |  |
| Postinterventional bleeding | T81.0 |  |  | Nachblutung; nachgeblutete |
| Flap necrosis | T86.51 |  |  | Lappennekrose |
| Laser techniques, CO₂ laser |  | 5-985, 5-985.1 |  |  |
| Robotic assistance |  | 5-987 |  |  |
| Radiotherapy |  | 8-52, 8-522, 8-523, 8-524, 8-525, 8-52a |  |  |
| Systemic antineoplastic therapy |  | 8-542, 8-543, 8-544, 8-547.0, 8-547.1 |  |  |

**Supplementary Table S1 (continued).**

| **Variable** | **Data source / operational definition** | | | |
| --- | --- | --- | --- | --- |
|  | **ICD-10-GM** | **OPS** | **Hospital information system** | **Terminology-based extraction:** **exact search concepts (German)** |
| **Peritherapeutic events** | | | | |
| Complex intensive care treatment |  | 8-980 |  |  |
| Transfusion of cellular blood components |  | 8-800, 8-800.c |  |  |
| Surgical closure of a pharyngocutaneous fistula |  | 5-294.1 |  |  |
| Newly performed tracheostomy |  | 5-311, 5-312 |  |  |
| Tracheostomy-related documentation | Z93.0, Z43.0, J95.0, R13.1 |  |  |  |
| **Comorbidities and substance-related factors** | | | | |
| Arterial hypertension | I10-I15 |  |  | Hypertonie; Bluthochdruck; Hypertonus; Blutdruckerhöhung; hypertensive; Hypertension |
| Diabetes mellitus | E10-E14 |  |  | Diabetes; Diab; diabetische |
| Overweight- or obesity-related documentation | E66 |  |  | Adipositas; adipöse; Übergewicht; übergewichtige |
| Nicotine- or tobacco-use-related documentation | F17.- |  |  | Nikotin; Tabak; Nikotinabusus; Nikotinmissbrauch; Tabakmissbrauch; Tabakkonsum; Zigarettenkonsum; Zigarrenkonsum; Raucher; Zigarettenraucher |
| Alcohol-related documentation |  |  |  | Alkohol; Alkoholkonsum; alkoholische; Alkoholeinfluss; C2; alkoholbedingte; alcoholica; äthyltoxische |
| Alcohol withdrawal syndrome with delirium | F10.4 |  |  |  |
| **Morbidity indicators** | | | | |
| Hemoglobin levels at admission and nadir during hospitalization |  |  | Direct extraction |  |
| Duration of mechanical ventilation |  |  | Direct extraction |  |
| Length of hospital stay |  |  | Direct extraction |  |
| In-hospital mortality |  |  | Direct extraction |  |

**Note.** Terminology search concepts are reported in the original German wording used for German-language EHR documentation. Within each cell, the first listed term represents the primary search concept and the subsequent terms represent the supplied synonyms. The routine extraction engine additionally applied approximate string matching based on Levenshtein distance to capture minor spelling and word-form variants. Automated negation or contextual assertion detection was not implemented.

All terminology-based searches were restricted to the following predefined narrative EHR sections: discharge summaries ("Diagnoses" and "Progress/Summary" sections); ward-round documentation; nursing documentation; intervention/surgical reports ("Diagnoses" and "Surgical Report" sections); diagnostic imaging (X-ray/CT/MRI/ultrasound, Findings/Assessment section); pathology (Findings/Assessment section); and intensive-care documentation (ward-round and nursing documentation).

The alcohol-related terminology search was analyzed separately from alcohol withdrawal syndrome with delirium, which was identified exclusively using ICD-10-GM code F10.4.

“Direct extraction” indicates variables obtained directly from structured hospital information systems. Blank cells indicate that the respective source was not used for that variable. EHR, electronic health record; ICD-10-GM, German Modification of the International Classification of Diseases, 10th Revision; OPS, German Operation and Procedure Classification.
