# Supplementary material for: Inpatient morbidity and structural care burden in oropharyngeal carcinoma in Germany: a nationwide cross-institutional EHR analysis using an AI-enabled data network
Source: Eur Arch Otorhinolaryngol. 2026 Sep 17;283(12):8121–31. doi: 10.1007/s00405-026-10583-4 (PMC13768223; doi:10.1007/s00405-026-10583-4)
Supplement: Supplementary file 2 [file 405_2026_10583_MOESM2_ESM.docx]

**Supplementary Table S2. Distribution of OPC hospitalizations across grouped principal ICD-10-GM diagnosis categories**

| **Grouped subsite category** | **Included ICD-10-GM codes** | **Hospitalizations, n** | **Percentage, %** |
| --- | --- | --- | --- |
| Base of tongue and vallecula | C01, C02.4, C10.0 | 3,398 | 24.4 |
| Palate | C05.1, C05.2, C05.8, C05.9, C09.1 | 1,222 | 8.8 |
| Tonsil and lateral wall of the oropharynx | C09.0, C09.8, C09.9, C10.2 | 4,007 | 28.8 |
| Posterior wall of the oropharynx | C10.3 | 290 | 2.1 |
| Overlapping sites of the oropharynx | C10.8 | 4,164 | 29.9 |
| Other or unspecified oropharyngeal and adjacent pharyngeal sites | C10.9, C14.0, C14.2, C14.8 | 844 | 6.1 |
| **Total OPC hospitalizations** | | **13,925** | **100.0** |

**Note.** Percentages are based on 13,925 hospitalizations and are reported to one decimal place. Counts refer to the predefined grouped principal-diagnosis categories.
